# Molecular detection and phylogenetic analysis of Orf viruses from goats in Jiangxi province, China

**DOI:** 10.3389/fvets.2024.1389185

**Published:** 2024-05-30

**Authors:** Zhibang Zhang, Xiaoyan Zhang, Ping Meng, Kang Feng, Jinxiang Gong, Ziyin Yang, Taotao Yang, Xingli Xu, Wenya Zheng, Pengcheng Li

**Affiliations:** ^1^College of Life Sciences and Resources and Environment, Yichun University, Yichun, Jiangxi, China; ^2^Shanxi Key Laboratory of Ecological Animal Science and Environmental Veterinary Medicine, College of Veterinary Medicine, Shanxi Agricultural University, Jinzhong, Shanxi, China; ^3^College of Veterinary Medicine, Shanxi Agricultural University, Jinzhong, Shanxi, China

**Keywords:** Orf virus, Jiangxi province, molecular detection, phylogenetic analysis, China, goat

## Abstract

Orf is a zoonosis caused by the Orf virus (ORFV), which is endemic in goats, sheep, and wild ruminants worldwide. Orf infection is prevalent in China, with outbreaks reported in several provinces. Currently, there is limited information available regarding the characterization of ORFV strains in Jiangxi province. This study investigated an acute outbreak of Orf that occurred in 2021 in a goat herd in the Jiangxi province of China. Clinical signs in this case included lesions on the lips, nose, and inside the mouth. The presence of ORFV was confirmed from tissue samples by polymerase chain reaction (PCR). The nucleotide sequences of the B2L and F1L genes were fully sequenced and used to construct phylogenetic trees. The results of this investigation identified the ORFV JXxy2021 as the cause of the outbreak. The phylogenetic analysis revealed that the ORFV strain JXxy2021 had the highest similarity to the ORFV strains GO and FJ-SL from the neighboring province of Fujian. This suggests that JXxy2021 was likely transmitted from Fujian province. The results have provided valuable information on the genetic characteristics of JXxy2021 and the endemic situations of Orf in China.

## 1 Introduction

Orf, also known as sore mouth or contagious ecthyma, is a highly contagious zoonotic disease caused by the Orf virus (ORFV) (1). The disease usually manifests as scabbed sores on the lips, tongue, nostrils, and oral mucosa (2). The lesions are proliferative but self-limiting, and fatal outcomes are rare (3). However, the mortality of Orf can reach 90% in young animals, due to nutrient deficiency and secondary infection by pathogenic microorganisms (4). Orf primarily affects domestic small ruminants, such as sheep and goats, and occasionally wild small ruminants like deer and camels (5, 6). The disease can be transmitted to humans either through direct contact with infected animals or by consuming contaminated animal products (7–9). The case of human-to-human transmission of Orf has also been reported recently (10). Orf is widespread in most sheep- and goat-raising countries in the world (11). The global Orf endemic has caused significant economic losses for livestock farmers and poses a threat to public health as well (12).

Orf virus (ORFV), the etiologic agent of Orf, is the prototype member of the Parapoxvirus genus of the Poxviridae family (13). Other members of the genus include bovine papular stomatitis virus (BPSV), pseudocowpox virus (PCPV), and parapoxvirus of red deer in New Zealand (PVNZ) (14). The genome of ORFV is a linear double-strand DNA of about 138 kb with a G + C content as high as 64%, encoding 132 putative genes (15). The ORFV genome has a highly conserved core region (ORFs 009-111) and two variable terminal regions (ORFs 001-008, ORFs 112-132). The ORF011 (B2L) and ORF059 (F1L) genes, which are located in the core region of the ORFV genome, are highly conserved and commonly used for phylogenetic analysis of ORFV (16). Here we report the diagnosis and phylogenetic analysis of ORFV strain JXxy2021 in Jiangxi province of China. The full-length sequences of B2L and F1L genes were obtained. Multiple sequence alignments were performed based on the B2L and F1L genes. Phylogenetic trees were constructed to determine the genetic relationship of ORFV strain JXxy2021 with reference strains.

## 2 Materials and methods

### 2.1 Clinical cases and sampling

In 2021, a suspected outbreak of Orf occurred on a black goat farm in Jiangxi province, Southeast China. Following the first case, the goats on the farm were successively infected over an extended period. Tissue specimens were collected by scraping the crusts from affected areas, specifically the lips and nostrils, of goats with typical pathological changes consistent with Orf infection. The scab samples were stored at −80°C and analyzed further.

### 2.2 DNA extraction, PCR amplification, and sequencing

Pior to DNA extraction, the collected specimens were pre-treated as described below. Four samples of scab were collected from the goats exhibiting symptoms of the disease. A small quantity of each sample was selected and combined into a single sample. The mixed sample was then triturated in sterilized phosphate-buffered saline (PBS) using a mortar and pestle. The homogenized samples were centrifuged at 5000 r/min for 5 min at 4°C. The supernatant fluids were collected and used for downstream DNA extraction. Genomic DNA was extracted from 200 μL of viral stocks using the TIANamp RAN/DNA Kit (TIANGEN, Beijing, China) according to the manufacturer’s instructions.

The polymerase chain reaction (PCR) was conducted to ascertain the presence of ORFV in the sample. Two pairs of primers targeting the entire open reading frame of the B2L and F1L genes of ORFV (Accession No. KX951407 and KX951408) were designed using the software Primer Premier 5. The sequences of the primers are as follows: B2L-F: 5′-atgtggccgttctcctccatc-3′, and B2L-R: 5′-ttaatttattggcttgcagaac-3′, F1L-F: 5′-atggatccacccgaaatcac-3′, and F1L-R: 5′-tcacacgatggccgtgacca-3′. The amplification reactions were performed in a 50 μL reaction volume consisting of 25 μL of 2 × Phanta Max Master Mix (Vazyme Biotechnology, Nanjing, China), 2 μL of each primer, 2 μL template, and 19 μL of distilled water. After pre-denaturation at 95°C for 3 min, 33 amplification cycles were carried out. The amplification process consisted of denaturation at 95°C for 15 s, annealing at 56°C for 15 s, and extension at 72°C for 60 s. This was followed by a final extension at 72°C for 5 min. The PCR products were analyzed on 1% agarose electrophoresis.

To get the full-length sequences of the B2L and F1L genes cloned in this study, the PCR products were purified using a Gel Extraction Kit (Thermo Fisher Scientific, Miami, USA) and were cloned into the TA/Blunt-Zero cloning Kit (Vazyme Biotechnology, Nanjing, China). The recombinant plasmids, TA/Blunt-Zero-B2L and TA/Blunt-Zero-F1L, were sequenced by Sangong Company (Shanghai, China). The sequences of B2L and F1L were deposited in the NCBI GenBank database under accession numbers OQ686991 and OQ686990, respectively.

### 2.3 Phylogenetic analysis

The ORFV strains in the NCBI GenBank that have the highest similarity to JXxy2021 were identified using the online BLAST program and were included in the phylogenetic analysis. The reference sequences were retrieved from the NCBI database, and the information for each sequence is listed in Table 1. The nucleotide sequences of B2L and F1L from ORFV strain JXxy2021 and the reference strains were used for phylogenetic analysis. Multiple sequence alignments were performed using the Clustal W method in DNASTAR software (v7.0). Phylogenetic trees were constructed by the neighbor-joining (NJ) method of MEGA 11 software, with 1,000 bootstrap test replications (17).

**Table 1:** B2L and F1L genes used in phylogenetic analysis.

Gene origin		Accession no.	Host specie	Country	Year
F1L	Guizhou	KP057582	goat	China	2010
	NP	KP010355	goat	China	2011
	FJ-SL	KC568409	goat	China	2012
	GO	KP010354	goat	China	2012
	SJ1	KP010356	goat	China	2012
	YX	KP010353	goat	China	2012
	HuB13	KJ139993	goat	China	2013
	Xinjiang2	KF666561	goat	China	2013
	GDQY	KM583894	goat	China	2014
	AH1612	MF489130	goat	China	2016
	NA17	MG674916	goat	China	2016
	JXxy2021	OQ686990	goat	China	2021
	Jilin-Nongan	JQ271535	sheep	China	2011
	NA1/11	KF234407	sheep	China	2011
	OV-HN3/12	KC569751	sheep	China	2012
	SY17	MG712417	sheep	China	2016
	CL18	MN648219	sheep	China	2018
	SC	ON932451	sheep	China	2019
	Mukteswar Vaccine	KY412880	goat	India	2005
	MP	MT332357	goat	India	2017
	Mysore	KY412878	sheep	India	2010
	UPM/HSN-20	MW537048	goat	Malaysia	2018
	OV/7	AY040084	sheep	Italy	2001
	B029	KF837136	Home sapiens	Germany	1996
	D1701	HM133903	sheep	Germany	2010
	OV-SA00	AY386264	goat	USA	2003
	OV-IA82	AY386263	sheep	USA	1982
	TVL	MN454854	sheep	USA	2019
	NZ2	DQ184476	sheep	New Zealand	2005
B2L	NP	KP010355	goat	China	2011
	FJ-ZX	KC568400	goat	China	2012
	GO	KP010354	goat	China	2012
	SJ1	KP010356	goat	China	2012
	YX	KP010353	goat	China	2012
	NA17	MG674916	goat	China	2016
	JXxy2021	OQ686991	goat	China	2021
	NA1/11	KF234407	sheep	China	2011
	SY17	MG712417	sheep	China	2016
	CL18	MN648219	sheep	China	2018
	SC	ON932451	sheep	China	2019
	Mukteswar_passage9	ON380499	goat	Inida	2005
	ORFV-Hyderabad	MH790949	goat	India	2006
	MP	MT332357	goat	India	2017
	UPM/HSN-20	MW537048	goat	Malaysia	2018
	B029	KF837136	Home sapiens	Germany	1996
	D1701	HM133903	sheep	Germany	2010
	OV-SA00	AY386264	goat	USA	2003
	OV-IA82	AY386263	sheep	USA	1982
	TVL	MN454854	sheep	USA	2019
	NZ2	DQ184476	sheep	New Zealand	2005

## 3 Results

### 3.1 Case presentation

**Figure 1:**
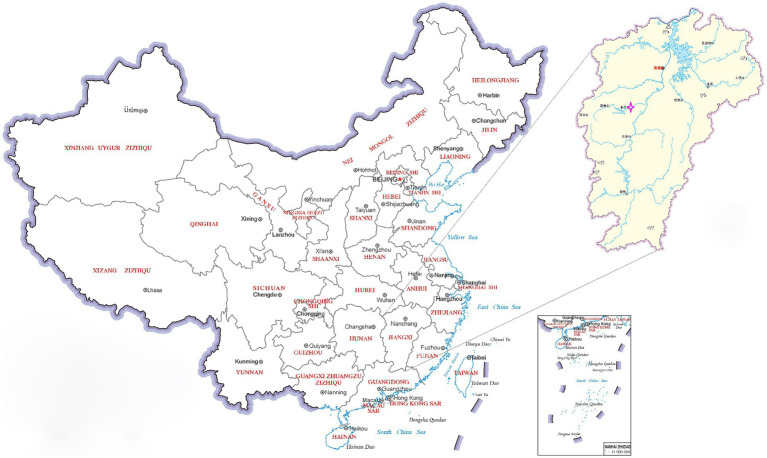
A map of China showing the sampling site. The pink four-pointed star 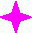 indicates the location of the outbreak of Orf. The map has been adapted from MAP WORLD (https://www.tianditu.gov.cn).

**Figure 2:**
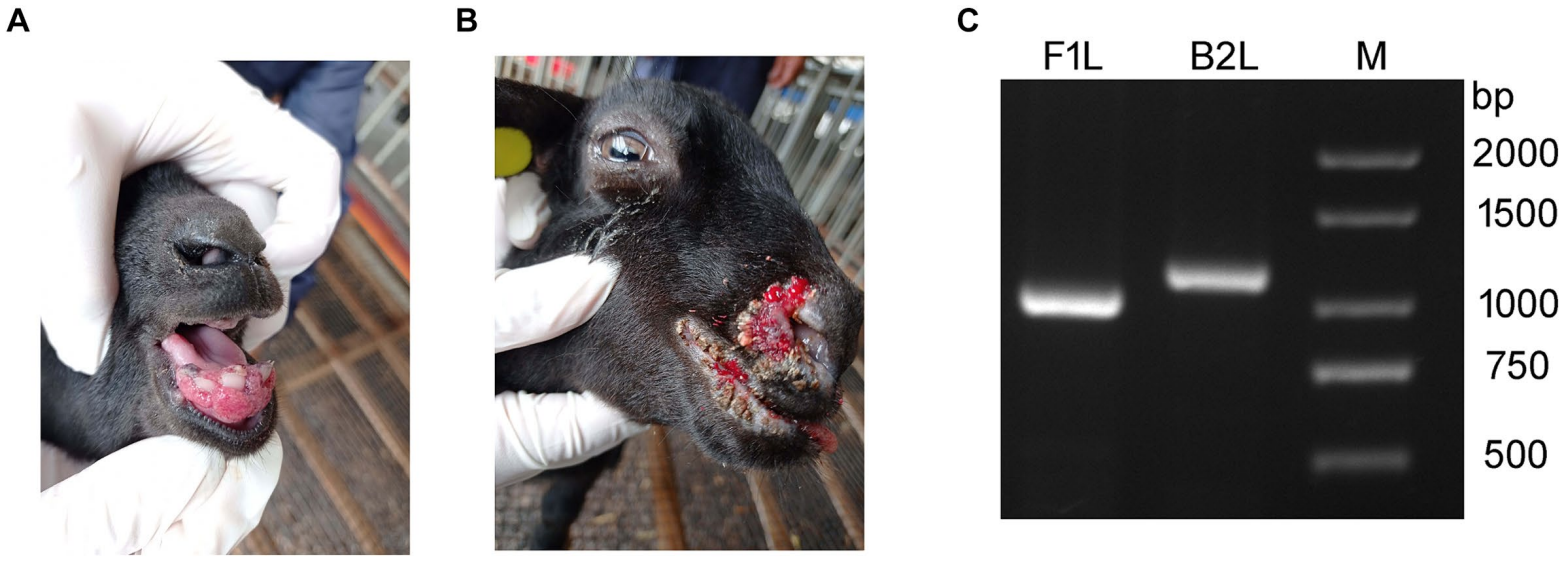
Representative clinical cases of ORFV infection in goat investigated using PCR. **(A,B)** Representative clinical cases of ORFV infection of goats. **(A)** Goat with severe lesions on the gum (Internal Orf). **(B)** Goat showing multiple nodular lesions on the lips and nostrils (External Orf). **(C)** PCR amplification of B2L and F1L genes using the DNA extracted from tissue samples. Lane M: DNA ladder.

In March of 2021, a suspected Orf outbreak was reported on a goat farm in Xinyu City, Jiangxi province (Figure 1). The goats that were suspected of having Orf first developed pimples and blisters on their lips, nostrils, gums, and tongue, which then led to ulceration (Figures 2A,B). This is consistent with the typical clinical manifestations of Orf (16). Over time, these sores tend to dry up and form a crust, but sores on the gums and tongue are more difficult to heal. Lesions around the lips, nostrils, tongue, and mouth can significantly impact a goat’s ability to feed, leading to a gradual decline in its physical and mental health. Based on these clinical signs, it was initially concluded that the goats were infected with the Orf virus (ORFV). The tissue samples were collected from the affected goats. The ORFV strain found in the samples was designated as JXxy2021 according to the time and location of the collection.

### 3.2 Confirmatory PCR

To confirm the preliminary diagnostic results, PCR was used to detect ORFV-specific genes, B2L and F1L, from the DNA extracted from the tissue samples. The PCR-amplified products were subjected to agarose gel electrophoresis. Two bands corresponding to B2L and F1L were observed in the agarose gel between 1,000 and 1,500 bp, consistent with the expected gene sizes (Figure 2). The results indicated that ORFV was present in the samples collected from the sick goats. This is the first time an ORFV infection has been detected on this farm.

### 3.3 Sequence determination

Identifying the origin and genetic characteristics of ORFV JXxy2021 is crucial as no previous literature has reported the presence of ORFV in Jiangxi province. To address these issues, it is necessary to determine the sequence of B2L and F1L genes of JXxy2021. These two genes are the most commonly used targets for phylogenetic analysis of ORFV (18, 19). For the sake of obtaining the complete sequences of B2L and F1L genes, we cloned the PCR products into the commercial TA-cloning vectors for sequencing. The sequencing results showed that the B2L and F1L genes have a full length of 1,137 bp and 1,029 bp, respectively. The two cloned genes, B2L and F1L, can be read without interruption as there are no additional stop codons, except for the ends of the sequences. The deduced proteins of the B2L and F1L genes are 378 and 342 amino acids, respectively. Taken together, the molecular diagnosis and clinical symptoms suggest an ORFV infection on the farm in this study.

### 3.4 Phylogenetic analysis

**Figure 3:**
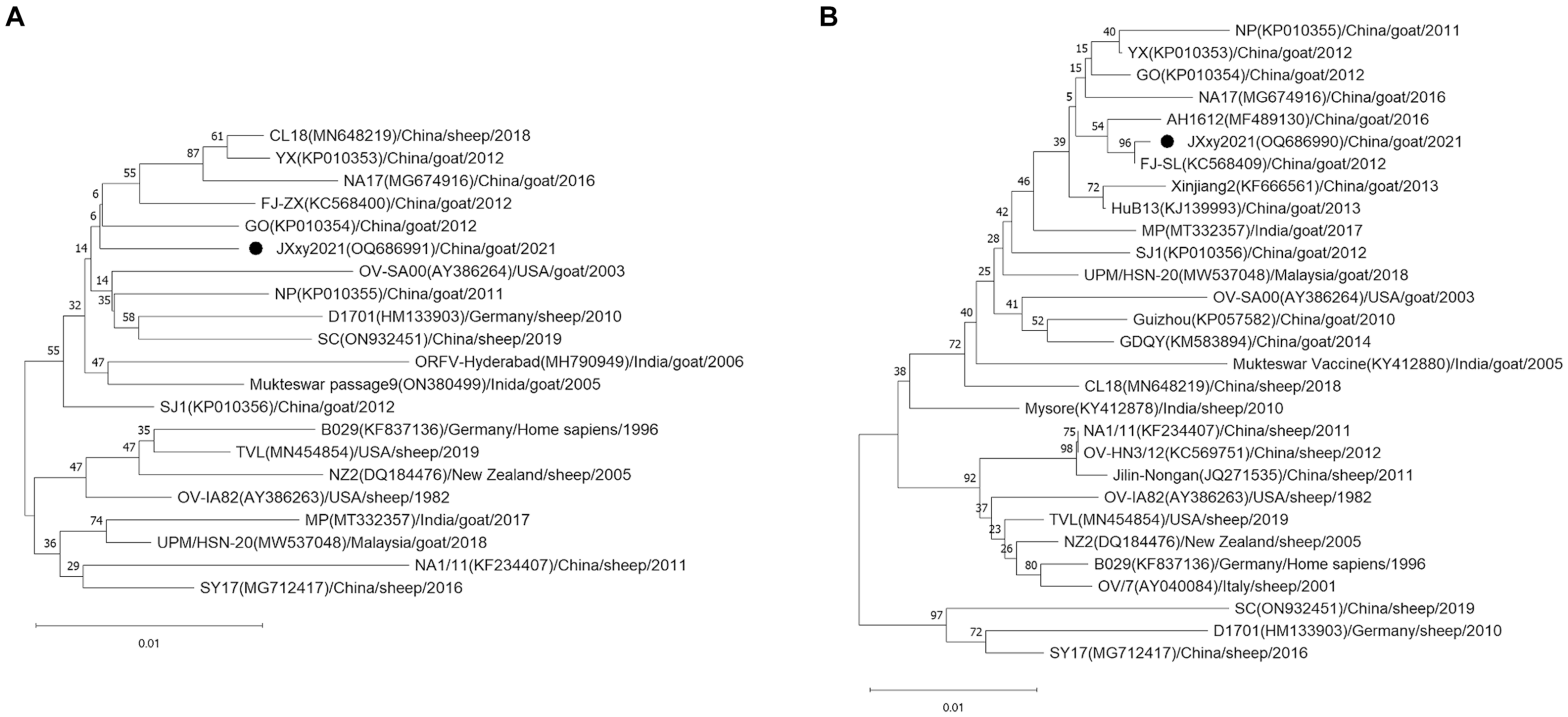
Phylogenetic trees based on the B2L **(A)** and F1L genes **(B)**. The B2L and F1L sequences obtained from this study are indicated by the black point ●. Each strain is presented with a taxonomic name, including the corresponding GenBank accession number, host, year, and country of origin. The trees were constructed using the neighbor-joining (NJ) method of MEGA 11 software with 1,000 bootstrap test replications.

To determine the phylogenetic relationship between JXxy2021 and other strains, we performed multiple sequence alignments of the B2L/F1L genes from JXxy2021 and reference strains. A phylogenetic tree was constructed by comparing the B2L nucleotide sequence with 20 reference strains obtained from the GenBank database. The result revealed that JXxy2021 shared the highest homology (98.8%) with the ORFV strain GO from Fujian province (Figure 3A; Supplementary Figure S1A). The phylogenetic tree, based on 29 F1L genes of various ORFV strains, indicated that the FJ-SL strain from Fujian province is genetically closely related to the JXxy2021 strain (Figure 3B), with a sequence identity of about 99.9% (Supplementary Figure S1B).

## 4 Discussion

Orf is a highly contagious zoonotic disease. The disease is common worldwide, especially in areas with high sheep or goat populations (20). China has the largest number of domestic small ruminants in the world (21). Orf is endemic in China, and several provinces have documented outbreaks of the disease (12, 22). The study reports an outbreak of Orf on a goat farm in Jiangxi province, Southeast China. The presence of Orf was detected through clinical signs and confirmed via molecular detection using PCR. In fact, the emergence of Orf on the farm occurred after breeding goats were introduced from the Sichuan province, Southwest China. It was initially hypothesized that Orf may have been caused by the introduction of breeding goats from outside. The ORFV strain SC was detected in a herd of sheep located in Sichuan province in 2017 (23). The nucleotide identity of the B2L and F1L genes between JXxy2021 and the SC strain is only 98.4 and 96.1% (Supplementary Figure S1), respectively. These values are not the highest among the results, indicating that the transmission of Orf from Sichuan province is less likely. Another possibility is that Orf was transmitted from the neighboring provinces to Jiangxi province. Jiangxi province shares borders with six provinces: Anhui, Hubei, Hunan, Guangdong, Fujian, and Zhejiang. Orf infection has been reported in four of the six provinces: Anhui, Hubei, Guangdong, and Fujian (22, 24–26). The phylogenetic trees based on the B2L and F1L genes indicate that the GO strain and FJ-SL strain have the highest homology to ORFV JXxy2021. Interestingly, GO and FJ-SL strains were discovered in Fujian province by the same research group (25, 27). The ORFV strains used for homology analysis in this study include those having the highest nucleotide sequence similarity to JXxy2021, which were identified through a BLAST search in the GenBank database. Based on the results obtained, JXxy2021 may have evolved from the ORFV strains found in the Fujian province. The findings indicate a potential cross-border transmission of ORFV due to close geographical proximity. However, the exact origin of the ORFV strain JXxy2021 remains unclear. Therefore, more epidemiological surveillance is needed in the future.

The B2L and F1L genes are regarded as epidemiologically relevant markers and are usually used in molecular epidemiological studies of ORFV (16, 25). The ORFV B2L gene encodes an envelope protein that is highly immunogenic and elicits a robust antibody response (28). The F1L protein is a key component of the microtubules found on the surface of ORFV and is responsible for stimulating the production of neutralizing antibodies in the host (29). Furthermore, the F1L protein is involved in the adsorption and invasion of host cells by the virus (30). However, the B2L and F1L genes are highly conserved in ORFV and may not accurately reflect the true genetic characteristics of different ORFV strains (31). Phylogenetic analysis based solely on conserved genes may overlook genetic heterogeneity among ORFV strains (32). Therefore, to accurately evaluate the genetic characteristics of ORFV strains, it is better to analyse more genes in addition to the B2L and F1L genes. However, the number of B2L and F1L genes of ORFV is the largest in the GenBank database. Currently, using the B2L or F1L genes as target genes for genetic evolutionary analysis of ORFV remains the dominant trend. Besides B2L and F1L genes, other genes, like ORF020 (VIR), GIF, vIL-10, ORF109, ORF110, ORF117, ORF119, ORF125, and ORF127 genes, have been utilized in the molecular epidemiological analysis of ORFV (6, 33–36). The ORF117, ORF119, ORF125, and ORF127 genes are located in the variable termini of the ORFV genome and are susceptible to mutation (37). Combining conserved and variable genes for phylogenetic analysis is more plausible than relying alone on conserved genes, which may introduce bias. This approach facilitates the acquisition of more comprehensive phylogenetic information on ORFV and enables better distinction between intraspecific strains (36).

In the view of some shepherds, Orf can be classified into two types: internal Orf and external Orf. If the lesions appear on the inside of the mouth, such as on the gums and tongue, it is considered as internal Orf. On the contrary, if Orf does not affect the inside of the oral cavity, it is referred to as external Orf. The healing process for lesions in the internal Orf is typically longer than that of the external Orf, resulting in a poorer outcome. The cases in this study (Figures 2A,B) happen to represent internal and external Orf, respectively. Similar situations can be observed in other cases and published literature (13, 24, 32). Currently, scholars may not yet be willing to accept the classification of Orf as internal or external based solely on clinical manifestations. The necessity and scientific validity of such a categorization should be discussed in the future.

This is the first report on the detection and phylogenetic analysis of an ORFV from Jiangxi province, Southeast China. This study identifies the genetic relatedness of JXxy2021 with other ORFV isolates from around the world. The results provide useful information on the endemic situations of Orf in China and may assist in preventing the transmission of Orf.

## Data availability statement

The original contributions presented in the study are publicly available. This data can be found at: https://www.ncbi.nlm.nih.gov/nuccore/; OQ686990-OQ686991.

## Ethics statement

The animal study was approved by the Animal Care and Ethics Committee of Yichun University (Permit No. JXSTUDKY2023130). The studies were conducted in accordance with the local legislation and institutional requirements. Written informed consent was obtained from the owners for the participation of their animals in this study.

## Author contributions

ZZ: Funding acquisition, Writing – review & editing, Writing – original draft, Investigation, Conceptualization. XZ: Funding acquisition, Writing – original draft, Methodology, Investigation, Data curation. PM: Writing – original draft, Software, Methodology, Investigation. KF: Writing – original draft, Validation, Investigation. JG: Writing – original draft, Validation, Software, Methodology. ZY: Writing – original draft, Resources, Methodology, Data curation. TY: Writing – original draft, Resources, Funding acquisition. XX: Writing – original draft, Supervision, Data curation. WZ: Writing – original draft, Supervision, Resources, Funding acquisition. PL: Writing – review & editing, Supervision, Funding acquisition.

## Supplementary material

SUPPLEMENTARY FIGURE S1 The percentages of identities and diversities of nucleotide sequences of the B2L **(A)** and F1L **(B)** gene among ORFV strains.
