# Supplementary figures and images for: Molecular detection and phylogenetic analysis of Orf viruses from goats in Jiangxi province, China
Source: Front Vet Sci. 2024 May 30;11:1389185. doi: 10.3389/fvets.2024.1389185 (PMC11188777; doi:10.3389/fvets.2024.1389185)

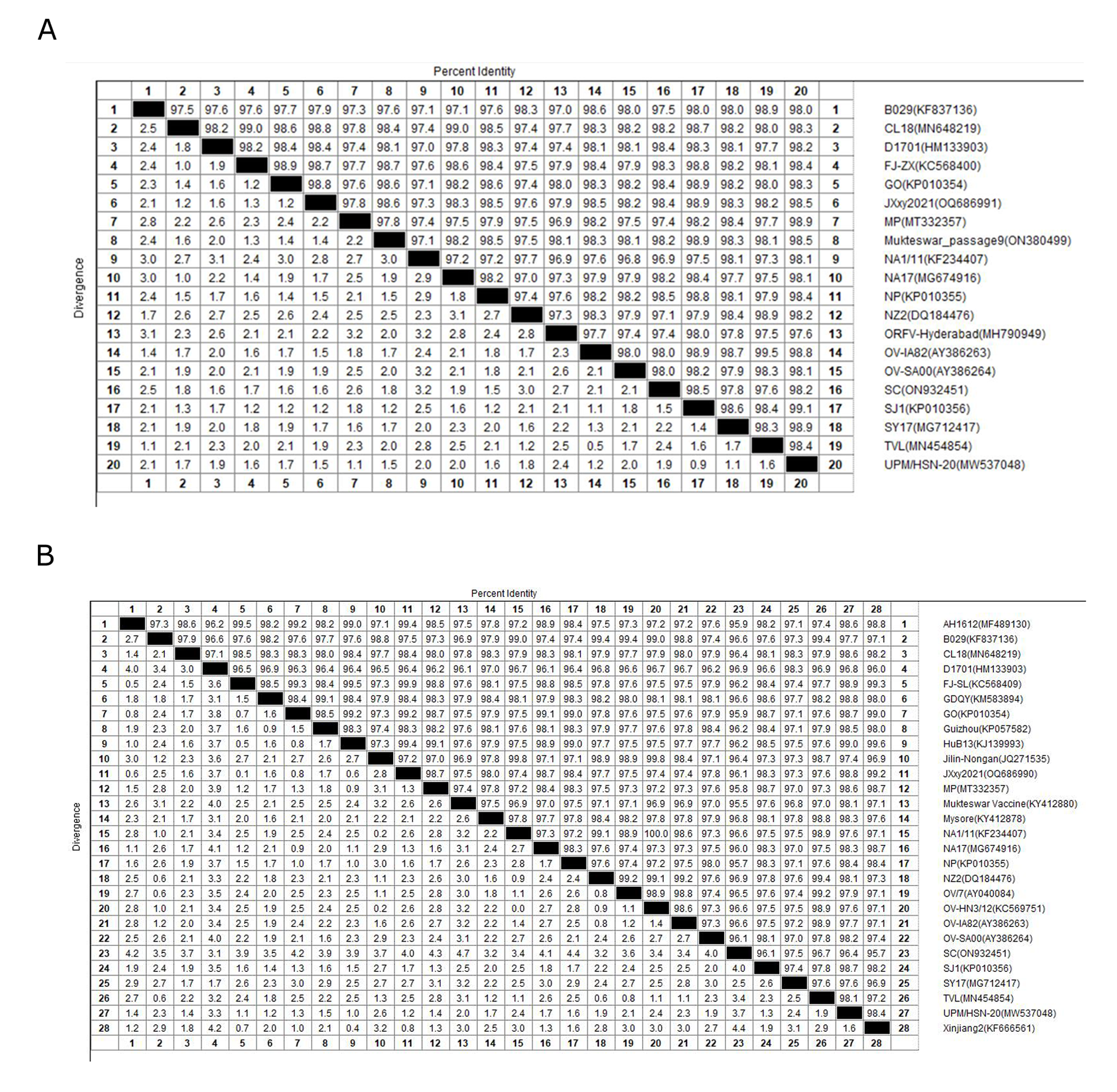

Supplement: SUPPLEMENTARY FIGURE S1 — The percentages of identities and diversities of nucleotide sequences of the B2L (A) and F1L (B) gene among ORFV strains. [file Image_1.tif]
